# Case report: a rare case of duodenal metastasis of endometrial carcinosarcoma

**DOI:** 10.3389/fonc.2024.1462029

**Published:** 2024-10-15

**Authors:** Lin Xiao, Lie Sun, Yi-Sheng Pan

**Affiliations:** Department of General Surgery, Peking University First Hospital, Beijing, China

**Keywords:** endometrial tumor, carcinosarcoma, duodenum, metastasis, epigastric pain

## Abstract

Endometrial carcinosarcoma is a tumor characterized by the coexistence of carcinoma and sarcoma. It almost only occurs in postmenopausal women, and the average five-year survival rate is less than 30%. Endometrial carcinosarcoma is very aggressive and usually has high tumor recurrence and mortality rates. Endometrial carcinosarcoma often metastasizes to the lymph nodes, lungs and peritoneum. Here, we report a rare case of duodenal metastasis of endometrial carcinosarcoma.

## Introduction

Endometrial carcinosarcoma is an aggressive high-grade endometrial carcinoma with secondary sarcoma dedifferentiation. Painless bleeding after menopause is a typical symptom; however, 15% of patients experience pelvic pain (1). As many as 30-40% of patients have lymph node metastasis at diagnosis, and 10% of patients have distant metastasis, among which, the lungs are the more common metastasis site (2). Metastasis of the poorly differentiated carcinomatous component of endometrial carcinosarcoma to the duodenum is very rare. There are no such case reports to date.

**Figure 1:**
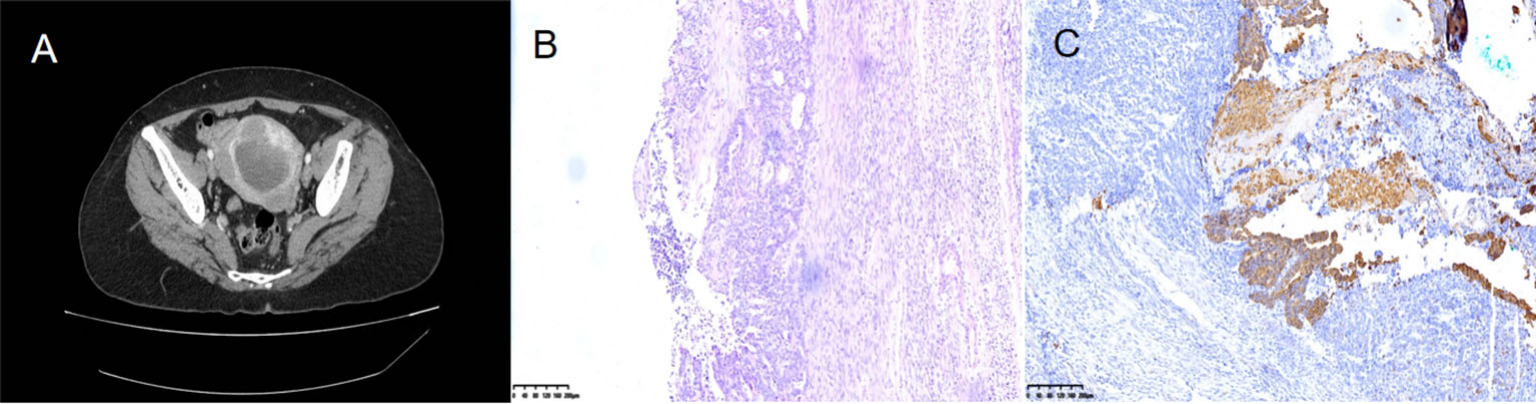
Contrast-enhanced computed tomography (CT) revealed a huge tumor in the uterine cavity **(A)**. Microscopically, adenocarcinoma and sarcoma were observed; the latter was similar to high-grade endometrial stromal sarcoma **(B)**. Immunohistochemical staining showed CKPan (AE1/AE3) (glandular epithelium+++/mesenchyme-) **(C)**.

**Figure 2:**
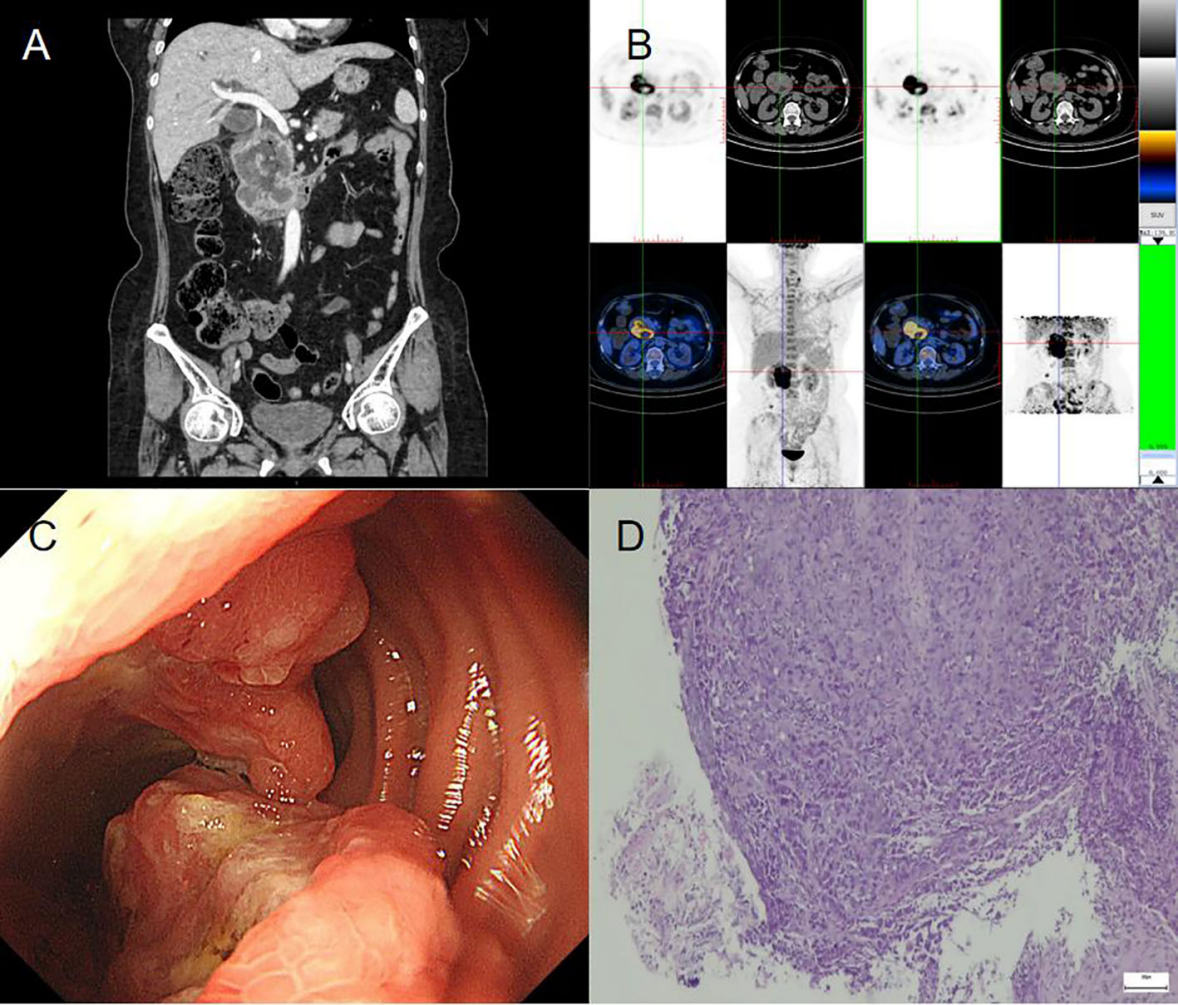
CT scan of the abdomen revealed a 4×5cm tumor in the descending duodenum **(A)**. Radionuclide imaging indicated the intestinal wall of descending duodenum thickening and increased glucose metabolism **(B)**. An electronic gastroscopy examination revealed a substantial tumor in the descending segment of the duodenum **(C)**. Biopsy results confirmed a poorly differentiated malignant duodenal tumor and showed epithelial differentiation characteristics consistent with the metastasis of poorly differentiated carcinomatous component in endometrial carcinosarcoma **(D)**.

The patient here was a 56-year-old woman who attended the Gynecology Department for painless vaginal bleeding after menopause. She denied a history of diabetes, hypertension or taking estrogen drugs. Tumor marker analysis revealed a carbohydrate antigen 125 level of 18.88 U/ml and a carbohydrate antigen 72-4 level of 0.89 U/ml. She had no family history of cancer. Contrast-enhanced computed tomography(CT) revealed a huge tumor in the uterine cavity (**Figure 1A**). Fractional curettage of the uterus revealed high-grade endometrial stromal sarcoma and adenocarcinoma components. After admission, the patient underwent a hysterectomy and bilateral salpingo-oophorectomy. The lymph nodes and omentum in the relevant areas were also completely removed. No tumor cells were found in the peritoneal lavage fluid during the operation. The postoperative pathology showed a tumor of 6×5×3 cm in size protruding from the uterine body and growing into the uterine cavity. Microscopically, adenocarcinoma and sarcoma were observed; the latter was similar to high-grade endometrial stromal sarcoma (**Figure 1B**). Immunohistochemical staining showed CKPan(AE1/AE3) (glandular epithelium+++/mesenchyme-) (**Figure 1C**). The patient was diagnosed with endometrial carcinosarcoma (stage II). Post-operatively, she received adjuvant chemotherapy with carboplatin and paclitaxel (TC regimen) and whole pelvic radiotherapy. After 1.5 years, she underwent gastrointestinal surgery for progressive epigastric pain with a loss of appetite. After admission, a CT scan of the abdomen revealed a 4×5 cm tumor in the descending duodenum (**Figure 2A**). Radionuclide imaging revealed thickening of the intestinal wall of the descending duodenum and increased glucose metabolism (**Figure 2B**). An electronic gastroscopy examination revealed a substantial tumor in the descending segment of the duodenum (**Figure 2C**). The biopsy results confirmed a poorly differentiated malignant duodenal tumor and showed epithelial differentiation characteristics consistent with metastasis of the poorly differentiated carcinomatous component of endometrial carcinosarcoma (**Figure 2D**). Although the patient only exhibited duodenal metastasis, the disease had entered the advanced stage. Because of her abnormal liver function and obvious abdominal pain, she could not be treated systematically. Ultimately, she underwent palliative surgery. Fortunately, the patient’s genetic test revealed high microsatellite instability (MSI-H). She was transferred to the Oncology Department for drug therapy.

## Discussion

Endometrial carcinosarcoma is a biphasic malignant tumor characterized by the coexistence of carcinoma and sarcoma (3). The epithelial component is the most important component and the sarcomatous component can be classified as homologous or heterologous according to whether the stromal component is similar to the uterine tissue. Heterogeneous differentiation is observed in about 40% of endometrial carcinosarcoma cases. Compared with homologous changes, heterogeneous differentiation is closely related to a poor survival rate (3). Endometrial carcinosarcoma accounts for about 5% of all uterine malignant tumors and nearly 20% of non-endometrioid endometrial cancers; 15% of deaths due to uterine malignant tumors are attributed to endometrial carcinosarcoma (4, 5). Importantly, the metastasis pattern of endometrial carcinosarcoma follows the lymphatic system and abdominal pathway, and its epithelial component is more prone to distant metastasis, while the sarcoma component is related to the local spread of the tumor (3). Sreenan et al. demonstrated that the recurrence and metastasis pattern of carcinosarcomas of the female genital tract is closer to carcinoma (6). The case described in this paper exhibited duodenal metastasis of the poorly differentiated carcinomatous component of carcinosarcoma.

Tumors in the duodenum are very rare, accounting for only 0.4% of all malignant tumors (7). Adenocarcinoma is the most common pathological type of primary duodenal tumor; it usually occurs in the second part of the duodenum. The most common tumor that metastasizes to the duodenum is malignant melanoma, followed by lung cancer (8). Cases of metastasis of female reproductive system tumors to the gastrointestinal tract are rare because most reproductive system tumors exhibit local expansion and lymphatic spread; hematogenous spread is rare (9). Chen et al. reviewed eight cases of cervical squamous cell carcinoma with duodenal metastasis. One case showed intestinal perforation, three cases showed intestinal bleeding, and the remaining cases experienced vomiting or persistent abdominal pain (10).

Endoscopy is an extremely important method to diagnose duodenal tumors. When an endoscopic examination identifies a duodenal tumor, it is necessary for an endoscopist to consider the most possible diagnosis and differential diagnosis. Doctors should pay attention to the patient’s past medical history to ensure that duodenal metastasis of other tumors is considered in the differential diagnosis process. Ultimately, a pathological biopsy is the gold standard for the diagnosis of duodenal tumors.

Historically, endometrial cancer was classified as uterine sarcoma. However, with progress in molecular research, it is now widely regarded as an endometrial epithelial dedifferentiation/metaplasia subgroup cancer. It originates from a single endometrial tumor clone undergoing metaplasia differentiation. Therefore, it should be more accurately regarded as an epithelial dedifferentiation tumor, and it should be staged and managed accordingly, as a high-grade endometrial cancer (11). In recent years, according to the risk of recurrence and progression-free survival, endometrial cancer has been reclassified and divided into the following four categories: DNA polymerase epsilon, microsatellite instability-high/defect mismatch repair (MSI-H/dMMR), TP53-wild-type, and TP53-mutant (12, 13). This molecular classification optimizes clinical management and employs specific analyses to determine the most appropriate and personalized auxiliary treatment methods.

According to the histological type, differentiation grade, surgical stage and molecular classification of endometrial cancer, endometrial cancer can be divided into low-risk, medium-risk and high-risk. Adjuvant therapy is not recommended for low-risk patients. Adjuvant brachytherapy can reduce vaginal recurrence in patients with moderate risk, and adjuvant pelvic external beam radiotherapy is recommended for patients with medium and high risks. High-risk patients have a high risk of recurrence. It is reported that chemotherapy and pelvic external beam radiotherapy should be performed at the same time or continuously after surgery (13). The pathological type of our case was carcinosarcoma, and the depth of muscle layer infiltration was more than 1/2 a layer. Thus, the patient was classified as high-risk and received chemotherapy and radiotherapy after surgery. With the clinical application of molecular typing of endometrial cancer, the treatment of endometrial cancer is becoming increasingly accurate, and the hierarchical management of cases is clear, which is positive for patients undergoing treatment.

The treatment of endometrial cancer primarily involves surgery. Radiotherapy and chemotherapy are common auxiliary treatments, with carboplatin and paclitaxel serving as the first-line treatment schemes (14–16). There is no strong evidence to support the use of the molecular spectrum in the postoperative treatment of patients with stage I-II disease. However, advanced cancer, mainly MSI-H/dMMR, may benefit from tailored postoperative treatment based on molecular analysis (12). Immune checkpoint blocking has been approved for the treatment of patients with MSI-H (17). The most famous immune checkpoint inhibitors are targeted programmed death ligand 1 (PD-L1) and programmed death -1 (PD-1) (18). The Food and Drug Administration approved pembrolizumab (humanized anti-PD-1 monoclonal antibody) as a monotherapy for metastatic or recurrent MSI-H endometrial cancer. The combination of pembrolizumab and lenvatinib is currently the second-line treatment for advanced/metastatic endometrial cancer after platinum-based chemotherapy (19, 20). Clinical studies on the use of immunotherapy combined with chemotherapy are currently underway. New molecular-targeted therapies (HER2-targeted drugs and WEE1 inhibitors) are currently being studied in prospective clinical trials. These may play a key role in the treatment of endometrial carcinosarcoma (14).

At present, although the treatment of endometrial carcinosarcoma is comprehensive, including surgery, platinum chemotherapy and radiotherapy, the prognosis of patients remains very poor. Even for patients who undergo surgery during stage I without adjuvant therapy, the five-year related mortality rate is about 50% (21). Surgery is not the first choice for the treatment of metastatic endometrial cancer. However, the current patient exhibited abnormal liver function and bilirubin. We concluded that this was caused by the compression of the bile duct by the duodenal tumor. Therefore, palliative surgery was carried out. On the seventh day after the surgery, the patient’s abnormal liver function and bilirubin returned to normal. The patient was discharged smoothly on the tenth day after surgery. Fortunately, her genetic test results showed MSI-H. We expect that the application of immunotherapy will bring good news to such challenging patients.

In summary, this paper reports a rare case of endometrial carcinosarcoma. Although the patient received comprehensive treatment including surgery, radiotherapy and chemotherapy, the poorly differentiated carcinomatous component still metastasized to the duodenum. The incidence of endometrial carcinosarcoma is slowly increasing, and the prognosis remains very poor. The rarity of endometrial carcinosarcoma, the lack of epidemiological research, the increasing incidence of endometrial carcinosarcoma, and the poor clinical prognosis highlight the need for new treatment strategies. Scholars should be focused on in-depth research on endometrial carcinosarcoma, so as to develop new targeted treatment methods and provide specific guidance for the future treatment of endometrial carcinosarcoma.

## Data Availability

The original contributions presented in the study are included in the article/supplementary material. Further inquiries can be directed to the corresponding author.
